# A Novel *Weissella cibaria* Strain UTNGt21O Isolated from Wild *Solanum quitoense* Fruit: Genome Sequence and Characterization of a Peptide with Highly Inhibitory Potential toward Gram-Negative Bacteria

**DOI:** 10.3390/foods9091242

**Published:** 2020-09-05

**Authors:** Gabriela N. Tenea, Pamela Hurtado, Clara Ortega

**Affiliations:** Biofood and Nutraceutics Research and Development Group, Faculty of Engineering in Agricultural and Environmental Sciences, Technical University of the North, Av. 17 de Julio s-21 Barrio El Olivo, 100150 Ibarra, Ecuador; pahurtadog@utn.edu.ec (P.H.); cgortega@utn.edu.ec (C.O.)

**Keywords:** lactic acid bacteria, de novo assembling, *Weissella cibaria*, antimicrobial peptides, *Escherichia coli*, *Salmonella* sp.

## Abstract

A novel *Weissella cibaria* strain UTNGt21O from the fruit of the *Solanum quitoense* (naranjilla) shrub produces a peptide that inhibits the growth of both *Salmonella enterica* subsp. *enterica* ATCC51741 and *Escherichia coli* ATCC25922 at different stages. A total of 31 contigs were assembled, with a total length of 1,924,087 bases, 20 contig hits match the core genome of different groups within *Weissella*, while for 11 contigs no match was found in the database. The GT content was 39.53% and the genome repeats sequences constitute around 186,760 bases of the assembly. The UTNGt21O matches the *W. cibaria* genome with 83% identity and no gaps (0). The sequencing data were deposited in the NCBI Database (BioProject accessions: PRJNA639289). The antibacterial activity and interaction mechanism of the peptide UTNGt21O on target bacteria were investigated by analyzing the growth, integrity, and morphology of the bacterial cells following treatment with different concentrations (1×, 1.5× and 2× MIC) of the peptide applied alone or in combination with chelating agent ethylenediaminetetraacetic acid (EDTA) at 20 mM. The results indicated a bacteriolytic effect at both early and late target growth at 3 h of incubation and total cell death at 6 h when EDTA was co-inoculated with the peptide. Based on BAGEL 4 (Bacteriocin Genome Mining Tool) a putative bacteriocin having 33.4% sequence similarity to enterolysin A was detected within the contig 12. The interaction between the peptide UTNGt21O and the target strains caused permeability in a dose-, time- response manner, with *Salmonella* (3200 AU/mL) more susceptible than *E. coli* (6400 AU/mL). The results indicated that UTNGt21O may damage the integrity of the cell target, leading to release of cytoplasmic components followed by cell death. Differences in membrane shape changes in target cells treated with different doses of peptide were observed by transmission electronic microscopy (TEM). Spheroplasts with spherical shapes were detected in *Salmonella* while larger shaped spheroplasts with thicker and deformed membranes along with filamentous cells were observed in *E. coli* upon the treatment with the UTNGt21O peptide. These results indicate the promising potential of the putative bacteriocin released by the novel *W. cibaria* strain UTNGt21O to be further tested as a new antimicrobial substance.

## 1. Introduction

In the last decade, a challenge for the food industry is to identify natural preservative compounds that will contribute to the overall increase of nutritional and health value when incorporated in foods, as well as to enhancing their safety and security [1]. High living standards have increased the demand for high-quality food, free of chemicals, thus new alternatives such as the incorporation of beneficial microorganisms or their metabolites in food might be a promising solution [1].

Several lactic acid bacteria (LAB) and their ribosomally synthetized peptides (bacteriocins), classified as “generally recognized as safe” retain very strong interest [2]. These molecules, naturally produced by bacteria living in a competitive microenvironment, are used to eliminate other bacterial species, particularly closely related ones [3]. This wide range of antimicrobial molecules secreted by LAB species allows a broad range of biotechnological, industrial, and pharmaceutical applications [1,2]. As conventional methods for conservation were proven to be unsafe for fresh food matrices, the use of LAB antimicrobial bacteriocins may be a suitable alternative to be investigated [4]. Although many LAB species were identified and characterized for their potential to protect against food poisoning, not all strains were found to be efficient, therefore, searching for new candidates with highly inhibitory spectra towards several multidrug resistant pathogens remains of interest. The killing activity is strain- and species-dependent and the inhibitory mode of action suggests a disruption of the bacterial membrane integrity, pore formation, and membrane permeability, leading to cell death [5,6]. With the rapid progress of sequencing technologies several LAB strains were sequenced, and genome assembled, to explore their diversity and applications. Among them, the genome of several species from genera *Weissella* was recently investigated [7,8]. Along with the probiotic capacity, some strains produced bacteriocins with a narrow antimicrobial spectrum [9,10]. Expanding the research on proper cataloguing of probiotic taxa with novel beneficial properties is an important task. Thus, recently we investigated the microbiota of several fruits and flowers from distinct subtropical native niches of Ecuador to select species with highly antimicrobial capacity [11,12,13]. *Solanum quitoense* L. (naranjilla) is a native plant of the Andes, cultivated and consumed mainly in Ecuador, Colombia, and Central America [14]. The plant grows between 1200 and 2300 m above the see level, especially under understory conditions; it grows in cool, shaded sites throughout Ecuador. It has an attractive bittersweet taste and drew our attention, as its microbiota had not yet been investigated. Numerous LAB isolates were selected and tested for their antimicrobial capacity against four Gram-negative bacteria found in local food: *Escherichia coli*, *Salmonella* sp., *Shigella* sp. and *Enterobacter* sp. [12]. In compliance with this stringent condition, among several isolates, the isolate with the code UTNGt21O, was identified. In this study, NGS de novo sequencing was performed to classify the species, a task that was not possible solely using 16 S rRNA [15]. Moreover, the mode of action of the peptide produced by UTNGt21O applied in different doses against *Salmonella enterica* subsp. *enterica* ATCC51741 and *E. coli* ATCC25922 was investigated by studying a series of cell biological analyses such as membrane permeabilization, target shape cell changes, and membrane integrity. This study will provide important information about the effectiveness of a peptide produced by a novel *Weissella cibaria* strain UTNGt21O to exhibit significant antimicrobial activity towards two Gram-negative bacteria, and the disclosure of a putative bacteriocin through genomic analysis.

## 2. Materials and Methods

### 2.1. Bacterial Isolation and Selection

The fruits were collected from a spontaneously growing plant found near the Amazonian jungle (Sucumbios Province, Ecuador) at about 326 m above the see level (unexpected growing environment). In brief, approximately ten grams of mature fruits sample were transferred into Erlenmeyer flasks (500 mL) containing sterile water (100 mL) and incubated statically for up to 5 days at room temperature. The LAB were selected on MRS agar plates upon incubation under anaerobic conditions at 37 °C for 72 h, as previously described [11]. The purified colonies (>100 colonies) were Gram stained, tested for mobility, indole-, catalase-production, spore formation and gas production from glucose. The selected colonies were tested for their capacity to inhibit four pathogenic bacteria found in local food: *E. coli*, *Salmonella* sp., *Shigella* sp. and *Enterobacter* sp. [12]. Among the isolates showing inhibitory activity, the isolate with the code UTNGt21O was selected and further sequenced.

### 2.2. NGS de Novo Sequencing of UTNGt21O Isolate

NGS de novo sequencing and assembly service was used (Ilumina HiSeq X Ten, Macrogen Inc., Seoul, Korea). For library construction, DNA/RNA was extracted from the sample (Illumina DNA prep kit, Illumina Inc., San Diego, CA, USA), checked for quality control and proceeded with the library construction (TruSeq DNA Nano kit, Illumina. Inc., San Diego, CA, USA). The sequencing library was prepared by random fragmentation of the DNA or cDNA sample, followed by 5′ and 3′ adapter ligation according to the protocol provided by the manufacturer (Macrogen Inc., Seoul, Korea). Adapter-ligated fragments were PCR amplified and gel purified. For cluster generation, the library was loaded into a flow cell where fragments were captured on a lawn of surface-bound oligos complementary to the library adapters. Each fragment was amplified into distinct clonal clusters through bridge amplification or ExAmp cluster amplification (patterned flow cells) followed by sequencing and the generated raw data were analyzed. Illumina SBS technology utilizes a proprietary reversible terminator-based method that detects single bases as they are incorporated into DNA template strands. As all 4 reversible, terminator-bound dNTPs are present during each sequencing cycle, natural competition minimizes incorporation bias and greatly reduces raw error rates compared to other technologies. The result was highly accurate base-by-base sequencing that virtually eliminates sequence-context-specific errors, even within repetitive sequence regions and homopolymers. Sequencing data was converted into raw data for the analysis. The overall quality of reads generated by FastQC (v0.11.5, http://www.bioinformatics.babraham.ac.uk/projects/fastqc), total bases, total reads, GC content, and basic statistics were calculated. To reduce biases in analysis, adapter trimming and quality filtering were performed. Trimmomatic (v0.36, http://www.usadellab.org/cms/?page=trimmomatic) was used to remove adapter sequences. The quality of filtered reads, total bases, total reads, GC content and basic statistics were calculated. De novo assembly was performed by various k-mer using SPAdes. K-mer analysis was performed to provide information about coverage, heterozygosity, and estimated genome size (Jellyfish v2.2.10, http://www.genome.umd.edu/jellyfish.html). Using filtered reads, de novo assembly was performed using a De Bruijn graph assembler (http://qb.cshl.edu/genomescope/). The reads were split into multiple K-mers (Oligonucleotides with length “K”, K equal integer), which were further aligned with each other, and sequences were extended one base at a time based on overlap information of K-mers. A best k-mer was selected based on various statistics from assembly results (number of contigs, total bases of contigs, N50, etc.) and a best assembled sequence set was determined. The assembled genome was validated using mapping strategy and BUSCO analysis (https://busco.ezlab.org/, BUSCO version 3.0) [16]. The filtered reads were aligned against the assembled genome and their insert sizes was estimated for validation. BUSCO analysis was performed to evaluate genome assemblies based on evolutionarily informed expectations of gene contents. These sequence data and assembly were submitted to the NCBI Database (BioProject ID: PRJNA639289).

### 2.3. Peptide Preparation, Determination of Minimum Inhibitory Concentration (MIC) and Molecular Weight

The partially purified peptide preparation and MIC values were determined as previously described [17]. To estimate the molecular size, the tricine-SDS-PAGE method using RunBlue Bis-Tris protein gels (20%) and Dual Cool Mini vertical PAGE/blotting Systems (Expedeon, Abcam, Cambridge, MA, USA) was used as previously described. The sample protein was run with a mass stained marker (Takara, Clearly stained protein ladder, cat #3454, Takara Bio. Inc., Mountain View, CA, USA). The gel was stained with InstantBlue ready-to-use stain (Expedeon, Abcam, Cambridge, MA, USA) for 4 h and distained with a solution of 30% methanol (*v*/*v*) and glacial acetic acid, 10% (*v*/*v*) until the bands became clear.

### 2.4. The Effect of Different Doses of Peptide UTNGt21O Alone and in Combination with EDTA on Indicator Cells Viability at Two Growth Stages

The indicator bacteria cells of *Salmonella enterica* ATCC51741 and *E. coli* ATCC25922, were grown respectively, in tubes containing nutritive broth and Luria–Bertani (Difco, Detroit, MI, USA) medium. The effect of different doses of peptide UTNGt21O alone or in combination with an external permeabilizing agent was evaluated [18]. The peptide was added to the cell culture of indicator bacteria at the early (OD605 = 0.2 ± 0.3) and late logarithmic (OD605 = 0.7 ± 0.5) growth phase respectively, as follows: (a) 1× MIC; (b) 1.5× MIC; (c) 2× MIC, of peptide UTNGt21O alone; (d) 1× MIC of peptide UTNGt21O combined with 20 mM EDTA (Sigma-Aldrich Co. LLC, Saint Louis, MO, USA); (e) 1.5× MIC of peptide UTNGt21O combined with 20 mM EDTA; (f) 2× MIC of peptide UTNGt21O combined with 20 mM EDTA; (g) indicator bacteria with 20 mM EDTA (h) control: untreated cells. Incubation was performed at 37 °C for 6 h and the cell viability was determined using the plate-agar method (BD Difco plate count agar, Fisher Scientific Co. LLC, Hampton, NH, USA) at 1, 3 and 6 h. The results were analyzed by determining the Log reduction calculated as the difference between log10 (CFU) of the untreated cells (no peptides, no EDTA) and the treated cells (peptides added, EDTA or a combination thereof). Log reduction of >1 was considered with the most significant (*p* < 0.05) [19].

### 2.5. Permeation of Cytoplasmic Membrane

Cytoplasmic membrane permeabilization by the peptide UTNGt21O was investigated using ONPG (o-nitro-phenyl-L-D-galactoside, # N1127, Sigma-Aldrich Co. LLC, Saint Louis, MO, USA) as substrate [19]. Briefly, bacteria cells grown to logarithmic phase in LB (Luria Bertani, Difco, Franklin Lakes, NJ, USA) or nutritive broth (Difco, Franklin Lakes, NJ, USA) medium containing 2% lactose were collected by centrifugation at 6000× *g* for 3 min and washed twice with 10 mM sodium phosphate buffer (pH 7.5) and 100 mM NaCl. The peptide UTNGt21O (1×, 1.5×, and 2× MIC) was added to the bacterial suspension at optical density 1.0, incubated for 5 min at 37 °C, then ONPG with a final concentration of 30 mM was added to each cell suspension. As control for permeabilization, Triton X 100 (1%, *v*/*v*) and EDTA (20 mM) were used. The hydrolysis of ONPG to O-nitrophenol (ONP) was monitored at 415 nm. To distinguish between cytoplasmic enzyme release and peptides uptake to the cells, the bacteria were removed by centrifugation at 6000× *g* for 3 min, at 120 min and the supernatant was used to measure the β-galactosidase release. The release of o-nitrophenol (ONP) per minute per mL was determined and calculated as described [20].

### 2.6. Cell Membrane Integrity Assay

If the bacterial membrane is compromised, release of inner cellular constituents such as DNA/ RNA can be monitored by determining the absorbance at 260 nm or gel electrophoresis [18] The indicator bacterial suspensions of *E. coli* ATCC25922 and *Salmonella enterica* subsp. *enterica* ATCC51741 were grown overnight in appropriate broth culture media, harvested by centrifugation, and washed twice with 1× PBS (phosphate buffered saline, pH 7.5) as described [18]. In brief, the bacterial cells were treated independently with different doses of the peptide UTNGt21O as follows: (a) 1× MIC; (b) 1.5× MIC; (c) 2× MIC, and incubated for 24 h at 30 °C. As control, one flask was maintained with no peptides added. The DNA/RNA molecules were extracted with chloroform (1:1, *v*/*v*), precipitated with isopropanol and ammonium acetate (3M), washed with 75% ethanol, followed by electrophoresis in 1% agarose gel with ethidium bromide, running in 1× TBE (Tris-borate EDTA, pH 8.0) buffer (Sigma-Aldrich Co. LLC, Saint Louis, MO, USA).

### 2.7. Transmission Electron Microscope (TEM)

For TEM analysis, the exponential-phase *E. coli* ATCC25922 and *Salmonella enterica* subsp. *enterica* ATCC51741 (1 × 10^8^ CFU/mL) cells were treated with 1×, 1.5× and 2× MIC of the peptide UTNGt21O for 6 h at 37 °C following the protocol as previously described [21]. Ultrathin sections were prepared and coated on copper grids and stained with uranyl acetate (Sigma-Aldrich Co. LLC, Saint Louis, MO, USA) and lead citrate (Sigma-Aldrich Co. LLC, Saint Louis, MO, USA). The grids (10 random sections per treatment) were examined using the Tecnai G2 F20 transmission electron microscope (FEI Company, Hillsboro, OR, USA).

### 2.8. Statistical Analysis

All experiments were performed in triplicate; the results were expressed as mean ± standard deviation. Analysis of variance was applied with least significant difference (LSD with Bonferroni correction) to determine significant differences between the means (SPSS version 15.0, SPSS Inc., Chicago, IL, USA).

## 3. Results and Discussion

### 3.1. Genome Assembly and Taxonomic Classification

**Figure 1:**
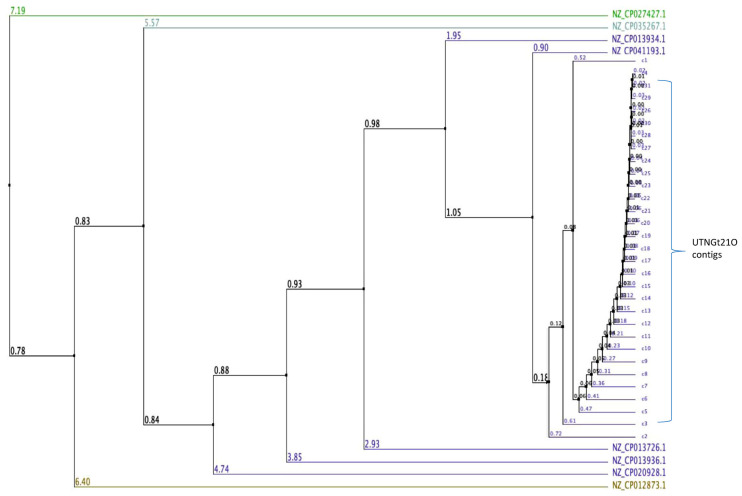
Average distance calculated based on percentage of similarity between sequences of some *W. cibaria* strains from data base and the total contigs of UTNGt21O. Legend: NZ_CP013936.1: *W. cibaria* strain CMU; NZ_CP013726.1: *W. cibaria* strain CMS2; NZ_CP041193.1: *W. cibaria* strain CBA3612; NZ_CP012873.1: *W. cibaria* strain CH2; NZ_CP020928.1: *W. cibaria* strain M2; NZ_CP027427.1 *W. cibaria* strain BM2; NZ_CP035267.1: *W. cibaria* strain SRCM103448; NZ_CP013934.1: *W. cibaria* strain CMS3. The number on branch is the bootstrap value that indicates the extent of relatedness between two subjects.

De novo genome sequencing is an important approach to underpin taxonomic, phylogenetic, evolutionary, and biodiversity studies to comprehensively decipher previously uncharacterized microorganisms [22]. Isolated from a wide range of environments such as fermented vegetables, wheat sourdoughs, feces, plants, lake water, the genome of several *Weissella* strains have been recently sequenced [7,8,23,24]. Only a few studies reported the narrow antimicrobial capacity of *Weissella* sp. [25,26], however understanding the inhibitory effect against commensal or pathogenic Gram-negative bacteria, and its mechanism of action remains limited. Previously, we inspected the microbiota of some tropical wild fruits and identified several LAB strains [11]. The predominant strains found in wild fruits belong to genera *Lactobacillus* and *Enterococcus*, while *Weissella* strains were barely found. For example, *W. confusa* strain Cys2-2 was found in wild spiral ginger with ability to suppress the growth of *E. coli* and *Salmonella* at the early growth stage [18]. In this study, the UTNGt21O strain isolated from wild fruit of *Solanum quitoense* was successfully sequenced and the genome assembled. The results indicated a total of 31 scaffolds with a total of 1,924,087 bases with 141.980 contiguity, with the largest contig of 422,230 bases and a minimum of 1435 bases. The GC content was 39.26% and Q30 was 86.81% (Table S1). The total number of bases, reads were calculated for the Gt21O sample after filtering (Table S2). The mean of K-mer coverage was 382.6, and the genome size was estimated at 2,044,333 bases with 186,760 bases of genome repeats length and 0.037 overall rate of heterozygocy. To validate the accuracy of the assembly, Illumina reads were mapped to the assembly result (Table S3). To assess the completeness of the genome assembly, BUSCO analysis was performed based on evolutionarily informed expectations of gene content from near-universal single-copy orthologs. The recovered matches were classified as complete if their lengths are within the expectation of the BUSCO profile match lengths. When found more than once, they were classified as duplicates. The matches that were only partially recovered were classified as fragmented, and BUSCO groups for which no matches pass the tests of orthology were classified as missing. Table 1 showed the results of BUSCO analysis. Higher complete BUSCOs indicate good assembly. However, for species other than model organisms, relatively low BUSCOs can appear due to the characteristics of the sample as well as the incompleteness of the assembly. After the complete genome was assembled, BLAST analysis was carried out to identify to which species each scaffold showed similarity. Out of 31 contigs analyzed 20 contigs hits matched the core genome of different groups within *Weissella* and for 11 contigs (15,17,21,23,24,26–31) no match was shown in database (Table S4). The largest contig 1 of the subject query (UTNGt21O) hits *W. cibaria* genome with an 83 percentage of identity with no gaps (0). The result is shown in Table 2. Because the BLAST analysis is based on registered information, the assembly results matched with a relative species or an evolutionarily distant species due to sequence differences or errors that may occur during the assembly process. Therefore, it was more appropriate to use the analysis results to identify patterns rather than to use it as an absolute criterion for species determination. Moreover, using a multiple genome sequence alignment with Jalview (version 2.10.1) [27], some *W. cibaria* strains retrieved from the GeneBank database and UTNGt21O total contigs (31) were compared and the average distance was calculated from the percentage of identity between the sequences, revealing a larger genetic variability within the strains (Figure 1). This analysis placed the UTNGt21O strain in the same node with NZ_CP041193.1 (*W. cibaria* strain CBA3612) and different clades than the NZ_CP027427.1 *W. cibaria* strain BM2. The sequencing data were deposited in the NCBI Database (BioProject accessions: PRJNA639289; BioSample accessions: SAMN15230453).

### 3.2. Identification of Putative Gene Cluster Encoding Bacteriocins

**Figure 2:**
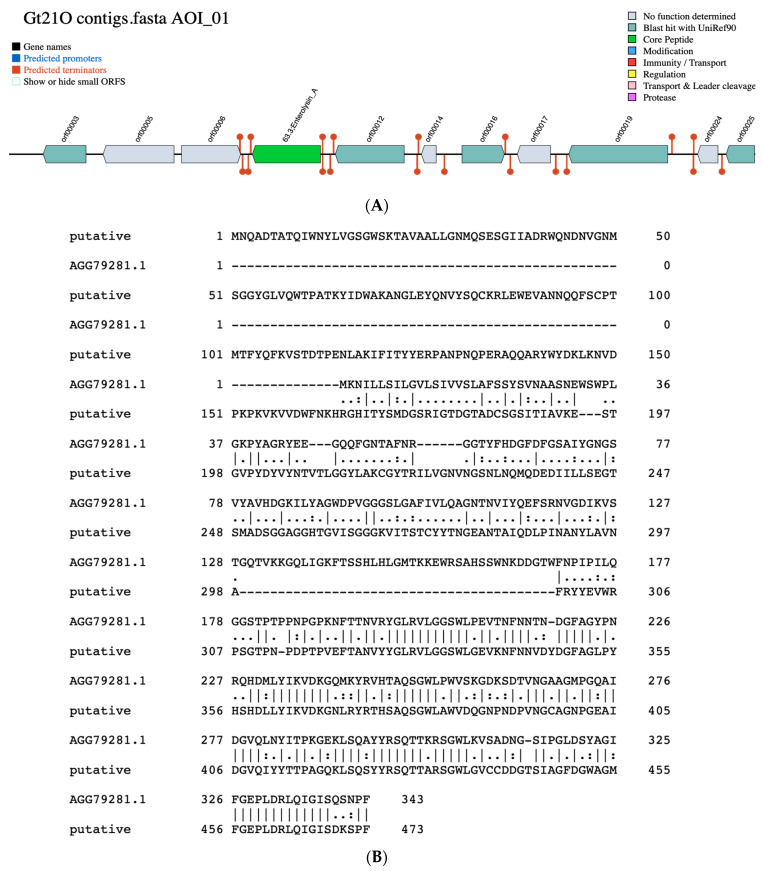
(**A**). Identification of a putative bacteriocin similar to enterolysin A and several proteins in UTNGt21O strain employing the BAGEL4 software (http://bagel.molgenrug.nl). AOI: area of interest (contig 12). (**B**). Protein sequence pairwise alignment of putative bacteriocin from UTNGT21O and enterolysin A from *Enterococcus faecalis* (NCBI accession no. AGG79281.1) with Clustal WS (v.2.0) retrieved from Jalview (version 2.10.1). The vertical lines showed the similar amino-acids.

By employing BAGEL 4 (Bacteriocin Genome Mining Tool), a freely accessible platform (http://bagel.molgenrug.nl) to identify genes which encoding bacteriocins or other unspecified ribosomally synthesized and post-translationally modified peptide product (RiPP) cluster [28] a bacteriocin with 33.4% sequence similarity to enterolysin A was found within the contig 12 (Figure 2A). The protein pairwise alignment showed the difference between the putative bacteriocin of UTNGt21O strain and enterolysin A from *Enterococcus faecalis*, having 27% sequence identity (Figure 2B). Enterolysin A is known as a cell wall degrading bacteriocin [29]. Among *Weissella* taxa the putative bacteriocin from UTNGt21O matches with 59.73% identity a hypothetical protein from *W. muntiaci* (Figure S1). Eleven ORFs sequences that encoding unknown proteins were also identified. Among the ORFs, five does not matches any bacteriocin/protein from the database, while orf00003, matches a manganese ABC transporter substrate binding lipoprotein of *Streptococcus* (28.73% species identity), orf00012 has similarity with catalase of *Lactobacillus sakei* (64.92% species identity), orf00016 has similarity with arylesterase from *Saccharolobus solfataricus* (30.14% species identity) and orf00025 matches a poly-beta 1,6-N acetyl-D-glucosamine synthase of *Staphylococcus aureus* (33.66% species identity), an exopolysaccharide important for bacterial aggregation and biofilm formation.

### 3.3. Peptide UTNGt21O Enhanced the Target Cell Lysis When Co-Cultivated with EDTA

**Figure 3:**
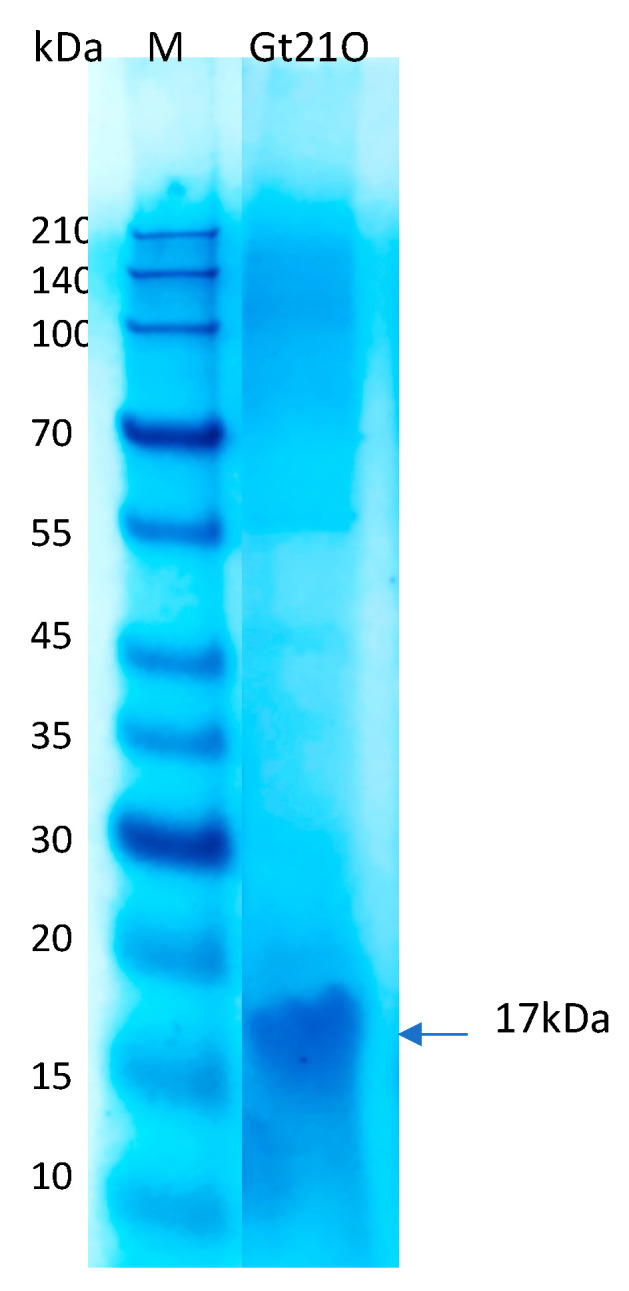
The molecular weight of UTNGt21O peptide extract deducted from SDS-PAGE analysis. M: molecular marker (Takara, Clearly Protein Ladder).

Natural antimicrobial peptides that inhibit the growth of pathogenic bacteria could be a suitable alternative to disinfectant solutions that are only meant to kill rapidly, but this feature is not maintained throughout time, such as conservation during storage [30]. In this study, to identify the effect of the peptide UTNGt21O on cell viability, different doses were added to *Salmonella enterica* subsp. *enterica* ATCC51741 and *E. coli* ATCC25922 cultures at two growth stages (early and late exponential phase) alone or co-inoculated with a membrane permeability agent (EDTA). The MIC was equivalent to 3200 AU/mL towards both target bacteria. The peptide molecular size estimated from SDS-PAGE analysis indicated a peptide/protein of about 17kDa (Figure 3). As previously observed, the size was larger than >10 kDa, as identified in other characterized bacteriocins from *Weissella* [23,25], and smaller than the 34kDa of enterolisyn A protein from *Enterococcus faecalis* strain LMG 2333 [29]. The cell viability of *Salmonella* decreased significantly (*p* < 0.05) at 3 h of incubation when the peptide was co-inoculated with the EDTA (8.22 log difference relative to untreated cells), while total cell death was registered at 2× MIC co-inoculated with EDTA and 6 h of incubation, suggesting that an extra agent enhanced the cell lysis of *Salmonella* by the peptide at the early growth stage (Table 3). Earlier research indicated that EDTA enhanced the reduction of Gram-negative bacteria cell viability when combined with bacteriocin [31]. Moreover, the addition of the peptide UTNGt21O in different doses resulted in a significant (*p* < 0.05) decrease of cell viability towards *E. coli* upon 1 h of incubation. No significative difference was observed between the doses tested and EDTA, while upon 3 h the cell viability diminished with 8 log difference than the untreated control, indicating the bactericidal effect of the peptide. The results suggest that the peptide killed *E. coli* without the need of an extra permeabilizing agent (Table 3). Previously, we showed that a peptide produced by *Lactobacillus plantarum* strain UTNGt2 diminished the viability of *E. coli* ATCC25922 but not *Salmonella enterica* subsp. *enterica* ATCC51741 without EDTA added [13]. In this study, there was no marked difference between the cell samples treated with EDTA and the untreated control. At early growth the cell viability diminished with 0.97 and 0.79 log and within 0.83 and 0.96 log at logarithmic growth for *Salmonella* and *E. coli*, respectively. The marginal effect of EDTA was previously reported with antibiotics against *Acinetobacter baumannii*, *Pseudomonas aeruginosa*, and *E. coli*, indicating that its effect cell lysis depends on the target [32]. At the late growth stage, the peptide UTNGt21O showed total *Salmonella* cell lysis at 3 h of incubation when co-inoculated with EDTA. No peptide dose response was observed. At 6 h, no cells were counted, indicating total cell death. Similarly, at early growth, EDTA alone had marginal effects on cell viability at the late growth. Early studies indicated that EDTA affected the outer membrane much more strongly in the early exponential phase than in the mid- or late exponential phase of *Salmonella* Typhi [33]. In case of *E. coli*, at the late exponential phase, a total cell death was registered at 6 h when treated with EDTA and high peptide dose. In this study, no viable cells of *E. coli* at the late growth stage were registered at the highest peptide concentration with EDTA. From a safety perspective, the combination of the peptide with EDTA was considered, as EDTA is the only food additive approved by the EU regulation no. 2008-1333 (E365) and has limited application in some foods and drinks according to Food and Drug Administration (FDA, Code of Federal Regulations 21 CFR 172.822). Thus, combining the bacteriocin with other existing preservatives will be a better option to enhance the killing effect. These results are in agreement with our previous findings indicating that the antimicrobial activity increased when cotreating the bacteriocin with EDTA as an increase of the bacteriocin solubility leading to inhibition of the target bacteria [12,18]. Branen and Davidson showed the efficacy of nisin, a bacteriocin produced by *Lactococcus lactis*, combined with EDTA against enterohaemorrhagic *E. coli* strains [34]. Additive effects against *Salmonella* serovar Typhimurium were observed when nisin was combined with EDTA or ampicillin while a time-killing effect was observed when nisin was combine with cefotaxime or ceftriaxone [35]. We conclude that the peptide UTNGt21O had the ability to kill both target microorganisms and the cell lysis depends upon the dose applied, co-treated with an extra destabilizing membrane agent as well as by the target growth stage, *E. coli* being sensitized at the early stage without the need of EDTA, while *Salmonella* kill-response was EDTA-dependent. At the exponential growth phase *E. coli* was lysed by the peptide UTNGt21O in combination with EDTA, while *Salmonella* was sensitized without the need of EDTA with cell death registered at 3 h.

### 3.4. Peptide UTNGt21O Permeated the Target Cell Membrane

**Figure 4:**
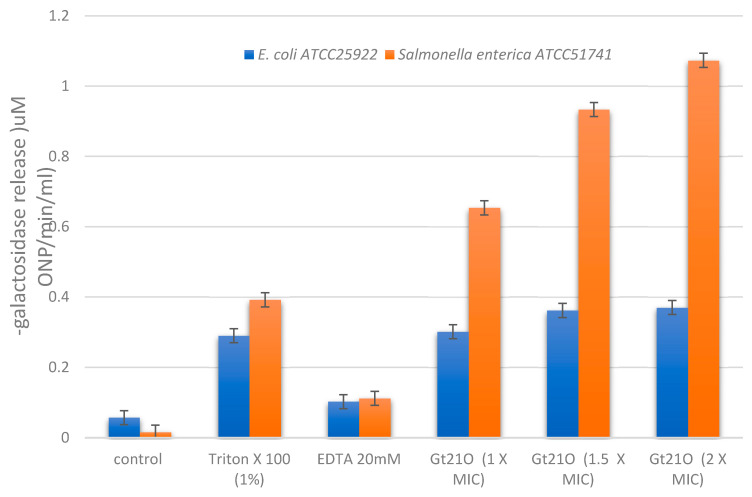
Permeation of *E. coli* ATCC25922 and *Salmonella enterica* subsp. *enterica* ATCC51741 by the peptide UTNGt21O upon 120 min of incubation by measuring the release of cytoplasmic β-galactosidase activity at OD415 nm. Legend: Control, cells treated with saline solution alone (no peptide added); EDTA, ethylenediaminetetraacetic acid. Results are representative of three similar and independent experiments each made in triplicate.

The ability of peptide UTNGt21O to permeate the membrane of *E. coli* and *Salmonella* was evaluated by determining the cytoplasmic β-galactosidase release in the cell-free medium. The peptide UTNGt21O displayed the ability to permeabilize the inner membrane to ONPG at all doses tested, while no activity was detected in the cells without any treatment. The peptide caused a considerable release of the enzyme into the medium within 120 min of incubation with *Salmonella* cells, while no significative release was detected with *E. coli* (Figure 4). No significant dose response was detected in case of *E. coli*, while with *Salmonella* cells a dose dependent response was detected, suggesting that with the increase of the peptide concentration the membrane destabilized increasing its permeability. When *Salmonella* cells were treated with 2× MIC peptide and the ONP release was monitored over time (30, 60, 80, 100, and 120 min), we observed that the steady-state level of β-galactosidase was recorded at 60 min and remained constant while in case of *E. coli* the steady state level was reached at 100 min (data not shown). However, the increase of cytoplasmic membrane permeabilization may correlate with the membrane disruption, leading to leakage of cell cytoplasmic content and cell death. Early studies of Miao et al., [20], indicated that the cytoplasmic membrane of *Staphylococcus aureus* was found to be the target for peptide F1 from *L. paracasei* subsp. *tolerans* FX-6 bactericidal activity due to membrane permeabilization in a kinetic manner. The results indicated that EDTA alone does not permeate the membrane, thus no direct killing effect occurs, while Triton-X100 showed a marginal effect (Figure 4). These results are in agreement with our previous research showing a narrow effect of Triton X100 on overall inhibitory activity towards *E. coli* [12].

### 3.5. Integrity of Target Cell Membrane Treated with the Peptide UTNGt21O

**Figure 5:**
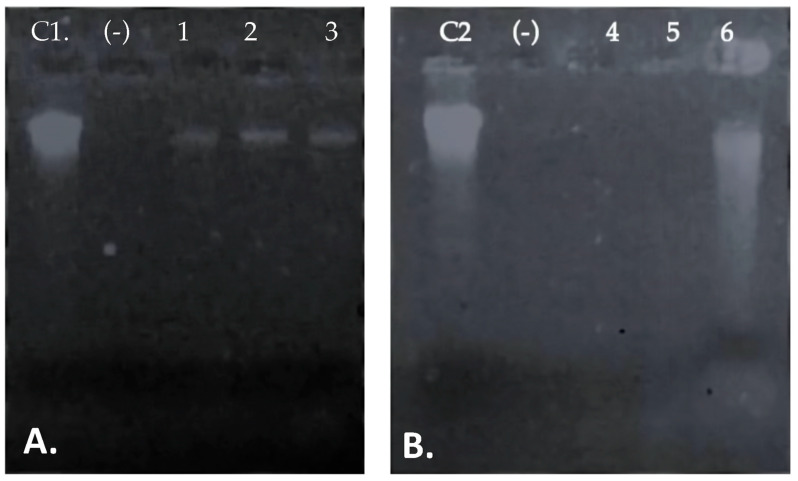
The effect of different dose of the peptide UTNGt21O on target membrane integrity. (**A**). *Salmonella enterica* subsp. *enterica* ATCC51741, (**B**). *E. coli* ATCC25922. Legend: C1, C2: genomic DNA of *Salmonella* and *E. coli*; (−): negative control (absence of DNA/RNA molecules in untreated cells); 1–3: DNA/RNA molecules detected upon 1×, 1.5×, and 2× MIC of UTNGt21O peptide. 4–5: absence of DNA/RNA upon the treatment with 1× and 1.5×, MIC of UTNGt21O peptide; 6-DNA/RNA detection upon 2× MIC of UTNGt21O peptide applied.

The outer membrane of Gram-negative bacteria is an essential cellular structure that serves as a selective permeation barrier [36]. However, if the cell membranes integrity is compromised, cellular molecules such DNA/RNA are released [37]. Figure 5 shows the effect of different doses of peptides on both *Salmonella* and *E. coli* cell membrane (Figure 5, lane C1, C2). The *Salmonella* DNA/RNA molecules were detected at all doses tested (Figure 5, lanes 1–3), while in case of *E. coli,* DNA/RNA molecules were detected at the highest concentration (2× MIC) of the peptide UTNGt21O upon 24 h of incubation (Figure 5, lane 4). The results suggest that to induce cell damage and leakage of cytoplasmic molecules, a high dose of peptide is required for *E. coli*, while the *Salmonella* membrane was more susceptible, and its alteration was not dose dependent. The results confirm our findings that the peptide UTNGt21O disrupts the cell membrane of the target bacteria. In addition, when bacteria are in their normal physiological state, external nutrients such as carbohydrates will be absorbed for proliferation, thus if the membrane is affected by the interaction with the peptide, a leakage of total soluble solids from cytoplasm might occur. Complementary analysis indicated a value of 9.4% and 5.6% Brix (the amount of dissolved solids in a liquid determined using a refractometer) detected in *Salmonella* and *E. coli* supernatant treated with 2× MIC peptide UTNGt21O, while in no treated peptide the registered values were 2.9 and 3.0% Brix, which might correspond to the growth medium carbohydrates. This result was consistent with the leaked cell content from bacterial cytoplasm indicating target-dependent interaction specificity and susceptibility. Thus, we conclude that the peptide produced by *W. cibaria* UTNGt21O in interaction with target bacteria might disrupt the normal cell membrane function.

### 3.6. The Peptide UTNGt21O Generate Changes of the Target Cell Membrane Shape

**Figure 6:**
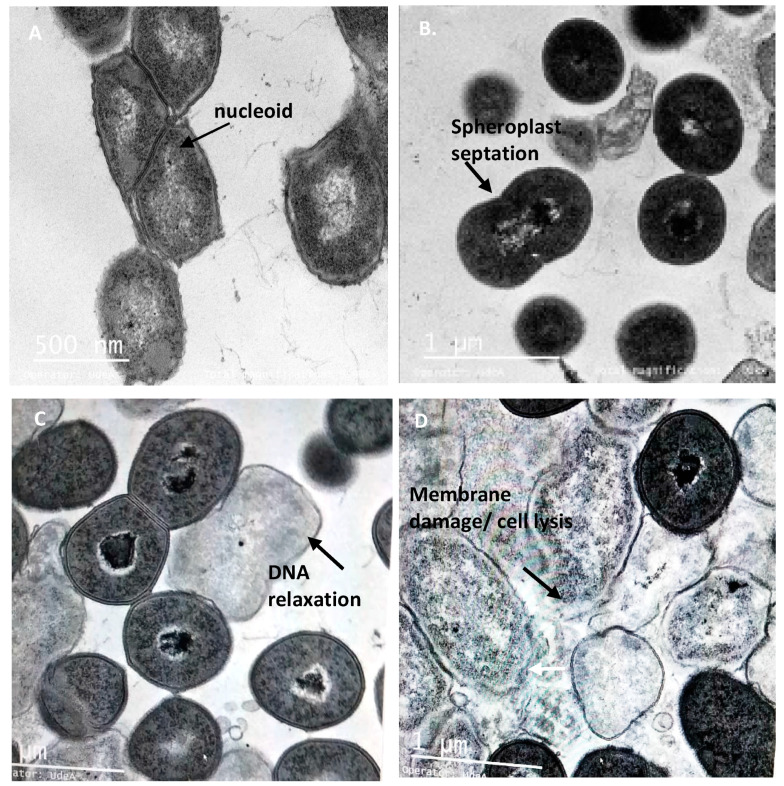
TEM micrographs of *Salmonella enterica* subsp. *enterica* ATCC51741 treated with the peptide UTNGt21O. Legend: (**A**) *Salmonella enterica* susbsp. *enterica* ATCC51741 untreated cells (exponential phase) in isotonic medium with visible and intact membrane and nucleoid structure; (**B**–**D**) *Salmonella* cells treated with 1×, 1.5× and 2× MIC of UTNGt21O peptide showing visible changes in cell morphology, with DNA relaxation, cytoplasmic condensation and spheroplasts with thicker membrane. Scale bars correspond to 500 nm (**A**) and 1 μm (**B**–**D**).

**Figure 7:**
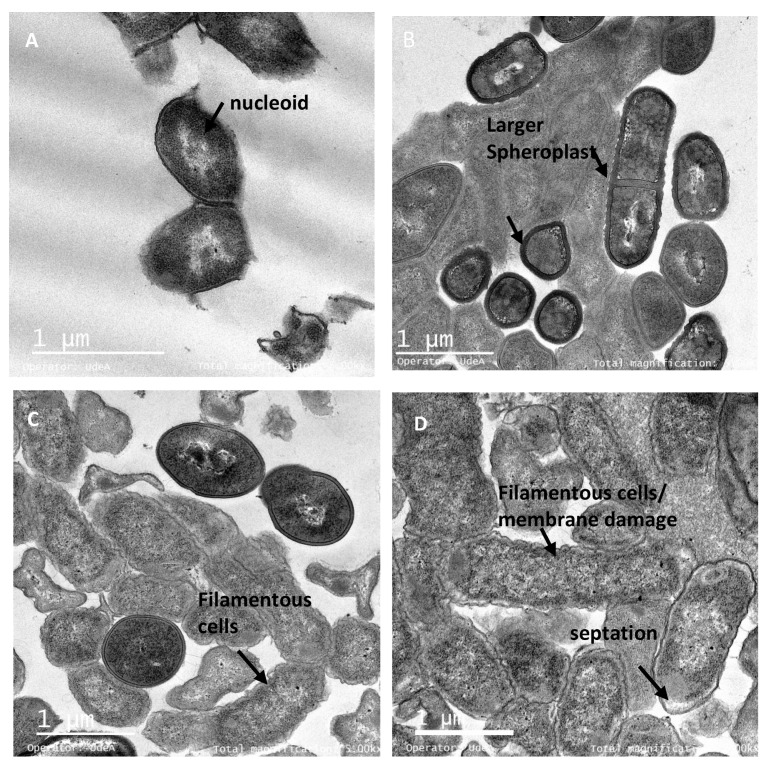
TEM micrographs of *E. coli* ATCC25922 treated with the peptide UTNGt21O. Legend: (**A**) *E coli* ATCC25922 untreated cells (exponential phase) in isotonic medium with visible and intact membrane and nucleoid structure; (**B**–**D**) *E coli* cells treated with 1×, 1.5× and 2× MIC of UTNGt21O peptide with visible changes in the outer and inner membrane, thick cell membrane and spheroplasts, larger filamentous cells and periplasmic space. Scale bars correspond to 1 μm.

Transmission electron microscopy (TEM) was performed on the bacterial cells after exposure to different doses of peptide to further understand the effect on the target cells shape. Untreated *Salmonella* cells at exponential growth showed normal shape with intact cell membrane and no noticeable ruptures or pores on the cell surface (Figure 6A). After treatment with different doses of peptide, the spheroplasts at 1× and 1.5× MIC had visible membrane disruption and at 2× MIC had the significant visible cell lysis. (Figure 6B–D). The spherical shape structure of bacteria is the effect of membrane tension that preserves its outer membrane but has lost the peptidoglycans layer. The production of spheroplasts upon *Salmonella* cells treated with a combination of peptides from LAB was recently observed [21]. The untreated *E. coli* cells were shaped with no damage to the structure of the plasma membrane or the outer membrane, and the cytoplasm appeared to have homogeneous electron density (Figure 7A). Upon 6 h of treatment with the peptide UTNGt21O, a dose response effect was observed with larger shaped spheroplasts having thicker and deformed membranes at 1X (Figure 7B) and filamentous cells, visibly notched in the outer and inner membrane, heterogeneous electron density cytoplasm and larger periplasmic space at the concentrations of 1.5× and 2× MIC (Figure 7C,D). Early studies of *E. coli* treated with different antibiotics indicated that the filamentation probably occurs if DNA synthesis is inhibited [38,39]. A bacteriocin produced by *W. confusa* A3 isolated from milk was found to induce damage in the cell membrane of *Bacillus cereus*, a Gram-positive bacterium, as shown by TEM analysis [40]. We conclude that peptide UTNGt21O induced changes in the membrane due to its different mode of action towards *E. coli* and *Salmonella*. These ultrastructural changes may explain the differences in the bactericidal capacity and the target innate defense mechanism. Nonetheless, further studies regarding the cytotoxicity of the peptide as well as its effect on other food spoilage microorganisms are required. In addition, in agreement with other authors, the effect of the peptides produced by lactic acid bacteria during food fermentation and their potential to improve safety of the consumer should be investigated [41,42,43].

## 4. Conclusions

De novo sequencing combined with the functional properties gave new insights into the biology of a species. The novel *W. cibaria* strain UTNGt21O produced a peptide with high capacity to inhibit the growth of *Salmonella* enterica ATCC51741 and *E. coli* ATCC25922 in a dose-, time-dependent manner. The presence of an extra-permeabilization agent (EDTA) enhanced the killing of *Salmonella* and *E. coli* in a target development stage appearance, *Salmonella* being more susceptible than *E. coli.* Exposure of target cells to the peptide UTNGt21O resulted in cell membrane disruption with the DNA/RNA molecule leakage. The interaction of the peptide with *Salmonella enterica* subsp. *enterica* ATCC51741 and *E. coli* ATCC25922 resulted in several ultrastructural changes of the membrane cell shape as depicted by TEM analysis. Overall, these results indicated that UTNGt21O peptide is a promising natural antimicrobial agent with high potential to inhibit multiple targets at different growth stages. Knowledge of the exact mode of action of *Weissella* peptides would potentially help to tailor-make peptide-formulations which in combination with other antimicrobials, will target simultaneously specific pathogens. Further, from food safety perspective, it will be important to expand the research on understanding the mechanism by which these antimicrobial peptides from *Weissella* act ex vitro, during food fermentation, as well as to test their efficacy on controlling the spoilage growth in different food matrices.

## Figures and Tables

**Table 1:** BUSCO analysis result.

Status	# of BUSCOs	Percentage
Complete BUSCOs (C)		
Complete and single-copy BUSCOs (S)	141	95.27%
Complete and duplicated BUSCOs (D)	1	0.68%
Fragmented BUSCOs (F)	1	0.68%
Missing BUSCOs (M)	5	3.38%
Total BUSCO groups searched	148	100.00%

Used Lineage: bacteria_odb9 (number of species: 3663, number of BUSCOs: 148) Status: A quantitative assessment list of the completeness in terms of expected gene content. The following two conditions are used to create a status: a. Expected range of scores; b. Expected range of length alignments. If both conditions are met, it is classified as Complete (These complete BUSCO matches are either single-copy or duplicated). If length alignments are not met, it is classified as Fragmented. If both conditions are not met, it is classified as Missing; # of BUSCOs: Identified count in sample; Percentage: Identified percentage in sample.

**Table 2:** Description of the best hits results matches in NCBI NT database.

Description	Query #	Query Length	I_Pct. (%)
CP027427.1 *W. cibaria* strain BM2 chromosome, complete genome	1	422,230	83
CP014332.1 *W. jogaejeotgali* strain FOL01, complete genome	2	22,017	87
CP029684.1 *Oenococcus sicerae* strain UCMA 15228 chromosome, complete genome	1	59,319	73
CP027563.1 *W. confusa* strain VTT E-133279 chromosome, complete genome	4	532,349	75
CP041193.1 *W. cibaria* strain CBA3612 chromosome, complete genome	2	194,652	81
CP033608.1 *W. hellenica* strain 0916-4-2 chromosome, complete genome	2	279,551	77
CP026847.1 *W. koreensis* strain WiKim0080 chromosome, complete genome	2	66,496	79
CP023501.1 *W. paramesenteroides* strain FDAARGOS_414 chromosome, complete genome	1	4177	95
CP026849.1 *W. koreensis* strain WiKim0080 plasmid pWKW_2, complete sequence	1	6178	84
CP028160.1 *Lactococcus lactis* subsp. *lactis* strain 14B4 chromosome, complete genome	1	13,239	80
CP022606.1 *W. cibaria* strain CMS1, complete genome	2	236,419	80
CP035746.1 *Leuconostoc mesenteroides* strain SRCM103460 chromosome, complete genome	1	24,997	89

Query #: number of the sequenced match; Query Length: Query sequence length; I_Pct. (%): Percentage of identical matches.

**Table 3:** Difference in cell viability of *Salmonella enterica* subsp. *enterica* ATCC51741 and *E. coli* ATCC25922 treated with different doses of the peptide UTNGt21O and untreated control.

Peptide UTNGt21O Dose/(+/-) EDTA	Difference in Cell Viability Relative to Control Untreated (LOGCFU/mL)											
	Early Growth (OD605 = 0.2 ± 0.3)						Logarithmic Growth (OD605 = 0.7± 0.5)					
	*Salmonella enterica* subsp. *enterica* ATCC51741			*E. coli* ATCC25922			*Salmonella enterica* subsp. *enterica* ATCC51741			*E. coli* ATCC25922		
	1 h	3 h	6 h	1 h	3 h	6 h	1 h	3 h	6 h	1 h	3 h	6 h
1× MIC	0.68a	5.14b	6.00	1.68b	8.02b	ND	0.29a	7.22	ND	0.42a	5.67c	5.60
1.5× MIC	0.68a	5.83c	6.75	1.85bc	8.24b	ND	0.38a	7.22	ND	0.34a	5.42bc	7.53
2× MIC	0.68a	7.08ef	6.45	2.03c	8.24b	ND	0.58a	7.22	ND	0.04a	5.67c	7.64
1× MIC + EDTA (20 mM)	0.79a	6.91d	ND	1.75bc	8.54c	ND	0.37a	ND	ND	0.39a	5.37b	6.02
1.5× MIC +EDTA (20 mM)	0.79a	7.97f	ND	1.91bc	8.54c	ND	0.56a	ND	ND	0.27a	5.46bc	ND
2× MIC + EDTA (20 mM)	0.79a	8.22h	ND	2.21d	8.54c	ND	0.63a	ND	ND	0.07a	6.13d	ND
EDTA (20 mM)	0.80a	0.90a	0.97	0.64a	0.67a	0.79	0.77a	0.83	0.83	0.87a	0.93a	0.96

ND: no cells detected; Values represent the log CFU/mL difference between each treatment and untreated control. The common small letters within a column are not significantly different (*p* > 0.05).

## Supplementary Materials

The following are available online at https://www.mdpi.com/2304-8158/9/9/1242/s1, Table S1: Raw data stats; Table S2: Filtered data stats; Table S3: Overall mapping stats. Table S4. Summary of the scaffold similarity. Figure S1. Blast tree view based on pairwise alignment of putative bacteriocin sequence from UTNGt21O and proteins from *Weissella* taxa.
