# Supplementary material for: A Novel Weissella cibaria Strain UTNGt21O Isolated from Wild Solanum quitoense Fruit: Genome Sequence and Characterization of a Peptide with Highly Inhibitory Potential toward Gram-Negative Bacteria
Source: Foods. 2020 Sep 5;9(9):1242. doi: 10.3390/foods9091242 (PMC7555684; doi:10.3390/foods9091242)
Supplement: Supplementary file 1 [file foods-09-01242-s001.zip › Supplementary files.docx]

**Supplementary files:**

A novel *Weissella cibaria* strain UTNGt21O isolated from wild *Solanum quitoense* fruit: Genome sequence and characterization of a peptide with highly inhibitory potential towards Gram-negative bacteria

Gabriela N. Tenea ^1 ;*^, Pamela Hurtado^1^, Clara Ortega^1^

**Table S1.** Raw data analysis

| **Library name Total read bases Total reads GC (%) Q20 (%) Q30 (%)** |
| --- |
| Gt21O 1,344,911,960 13,315,960 39.26 94.01 86.81 |

Legend: Library name : Sample’s library name; Total read bases : The total number of bases sequenced

Total reads : The total number of reads. For Illumina paired-end sequencing, this value refers to

the sum of read1 and read2; GC (%) : GC content; Q20 (%) : Ratio of bases that have phred quality score of over 20; Q30 (%) : Ratio of bases that have phred quality score of over 30

**Table S2.** Filtered data stats

| **Library name Total read bases Total reads GC (%) Q20 (%) Q30 (%)** |
| --- |
| Gt21O_trimmed 866,077,393 8,608,218 39.54 98.86 95.59 |
| Gt21O_reduced 305,472,158 3,036,138 39.53 98.86 95.59 |

Legend: Library name: Sample’s library name; Total read bases: The total number of bases sequenced; Total reads: The total number of reads. For Illumina paired-end sequencing, this value refers to the sum of read1 and read2. GC (%): GC content; Q20 (%): Ratio of bases that have phred quality score of over 20; Q30 (%): Ratio of bases that have phred quality score of over 30

**Table S3.** Overall mapping stats

| **Library name Total read Mapped reads Coverage (%) Depth Ins. Size (Std.)** |
| --- |
| Gt21O 3,036,138 2,992,481 (98.56%) 100.00 153.53 410 (412.53) |
| Total 3,036,138 2,992,481 (98.56%) 100.00 153.53 - |

Legend:  Library name : Sample’s library name; Total reads : Total number of reads; Mapped reads: Total number of mapped reads; Coverage (%): The percentage of mapped sited (>= 1x); Depth: Average mapping depth; Ins.size (Std.): The length between adapters and standard deviation of predicted length

UTNGt21O

**Figure S1**. Blast tree view based on pairwise alignment of putative bacteriocin sequence from UTNGt21O and proteins from *Weissella* taxa (NCBI data base).
